# Department of Public Health and Vital Statistics, State of Texas

**Published:** 1908-03

**Authors:** 


Department of Public Health and Vital Statistics,
State of Texas.

Austin, Texas, February 8, 1908.

*To the Physicians of Texas.*

Gentlemen: The purpose of this letter is to call your attention to the law enacted by the Twenty-seventh Legislature requiring physicians to make reports of all births and deaths of which they are cognizant to the county clerk within thirty days after its occurrence, under a *penalty* for failure to do so of *five dollars for each birth and of not less than five or more than fifty dollars for each death.* It is not my purpose to antagonize the members of the profession, but your duty in this matter is very clear, and if you do not realize it it will be necessary for me to place the matter in the hands of the county attorney. "A hint to the wise is sufficient."

I have had blank certificates of births and deaths printed at the State's expense and they are now in the hands of the county clerks, and can be obtained by the physicians without cost. They are there for your use and there is absolutely no excuse for non-compliance with this law. There has been a steady increase in the number of physicians that are reporting, but partial reports are far from satisfactory, and I intend to see that the recalcitrant brother is prosecuted for non-compliance with the law.

Trusting that you will realize your duty in this matter without the intervention of the law, I remain,

Yours sincerely and fraternally,

WM. BRUMBY,
State Health Officer.

---

THE undersigned desires information regarding any alleged recoveries or cures of inoperable or recurrent carcinoma of the mammary gland.

If any case or cases are known to anyone who reads this circular and can be authenticated by facts as to the history and condition prior to recovery and the length of time which has elapsed since recovery such information will be much appreciated and duly acknowledged.

Any well authenticated reports of recoveries from carcinoma located in other parts than the mammary gland will be welcomed.

Cancer paste cures, X-Ray cures, radium cures, or cures as result of surgical operation are not wanted.

Hearsay cases are not wanted unless accompanied by name and address of person who can give knowledge first hand.

Address,

HORACE PACKARD,
470 Commonwealth Avenue,
Boston, Mass.

